# Effects of the inclusion of coffee pulp silage in the diet on the performance and profitability of crossbred milk cows in the middle tropics

**DOI:** 10.1007/s11250-023-03451-4

**Published:** 2023-02-13

**Authors:** Dixon Fabián Flórez-Delgado, Richard de Jesús Gil-Herrera, Román Enrique Maza-Ortega

**Affiliations:** 1 Department of Animal Science, Faculty of Agricultural Sciences, University of Pamplona, Km 1 Way Bucaramanga, Pamplona, Colombia; 2 Doctoral Department, American University of Europe, Av. Bonampak, Sm. 6, Mz. 1, Lt. 1, 77500 Cancun, México

**Keywords:** Tropical forage, Ruminant metabolism, Milk production, Food supplement

## Abstract

The objective of this study was to evaluate the inclusion of coffee pulp silage (CPS) on the performance and profitability of crossbred cows in the middle tropics. The research took place at the Villa Marina Experimental Farm of the University of Pamplona, Pamplonita, Colombia. Four lactating multiparous bovines with a body weight of 380 ± 10 kg, 6 ± 0.03 years of age and a body condition of 3.8 on a scale of 1 to 5. The animals were distributed in a balanced 4 × 4 Latin square for residual effect. The experiment consisted of four subperiods of 21 days each, being the first 14 days of adaptation to the experimental diet and 7 days for taking samples. The base diet consisted of *Pennisetum* sp. forage, concentrated in a proportion of 0.2% of body weight, water, and mineralized salt at will. The inclusion of the CPS was included in levels of 4, 8, and 12% of the dry matter (DM) intake. DM intake parameters, nutrient digestibility, milk production and composition, blood parameters, and economic analysis were evaluated. Treatments were compared using orthogonal contrasts; contrasts were constructed in order to evaluate the effects of inclusion of CPS, and the linear and quadratic effects of level inclusion in the DM of the diet. For the variables which did not present inclusion of CPS effect but a linear or quadratic effect was significant, a Dunnett’s test was performed to identify whether a supplemented treatment differed from the control. Significance was considered at *P* < 0.05 and tendencies when 0.05 < *P* ≤ 0.10. The inclusion of CPS in the diet increases (*P* < 0.05) the voluntary intake in kg day^−1^ of DM, organic matter (OM), CP, ethereal extract (EE), non-fiber carbohydrates (NFC), digested OM (DM), neutral detergent fiber corrected for ash and protein (NDFap), DNDF and dietary NTD concentration, the total digestibility of DM and OM, and dietary DOM content. The production and chemical composition of milk was not affected (*P* > 0.10) by the inclusion of EPC in the diet. The inclusion of CPS increased (*P* < 0.05) the concentration of albumin and globulins in the blood. The inclusion of CPS in the diet improved the productive efficiency of dairy cattle. The inclusion of 4% CPS in the DM of the diet is a nutritional strategy, which improves the nutritional characteristics and the concentration of albumin and globulins in the blood of crossed dairy cows in the middle tropics and benefit: cost ratio without affecting milk production.

## Introduction

Forages constitute the basal feed for cattle in production systems. However, they present metabolic and dietary deficiencies. Therefore, forage cannot be considered a nutritionally balanced diet (Detman et al., 2014). Due to the above, balanced foods are used to offset these differences. However, the use of these balanced feeds has a strong impact on production costs, drastically reducing the profit margin of the primary producer (Flórez et al., 2018). This situation has led producers to consider new foods and nutritional alternatives and environmentally sustainable. It is considered that the brown pulp meets the nutritional, economic, and environmental characteristics that, through the silage technique, allow its conservation for feeding ruminants, especially in the production areas of this fruit, in addition to mitigating the contamination of water resources and soil that is caused by its inadequate final disposal (Flórez et al., 2020). The fruit processing process aims to obtain dry parchment coffee without affecting its quality, reducing losses during the process, and generating a variety of by-products (Puerta, 2006). The beginning of this process occurs with the collection of the ripe fruits, their pulping and classification, continuing with the removal of the mucilage and ending with the drying of the grain (Noriega et al., 2008). It is here, where by-products such as mucilage, husk, and pulp are obtained (Pajoy, 2017) that generally do not receive an adequate final disposal and are thrown into watersheds causing contamination (Ocampo et. al., 2017).

Of a kilogram of coffee, the husk and mucilage represent 56% of the grain (Torres et al., 2019). The shell, also called the pulp, weighs around 43.6% of the fresh fruit (Rodríguez & Zambrano 2010). The pulp is rich in pectins, caffeine, proteins, carbohydrates and polyphenols, and is a potential source of high value-added agroindustry (Murthy & Naidu 2012). Its use in ruminants occurs as a partial replacement for commercial feed or as an addition to the dry matter of the base diet. This substitution ranges from 20 to 40% or up to 20% of the dry base ration without affecting the productive performance of the animals. In the case of fattening cattle, the inclusion ranges up to 30% of the ration, since higher values affect dry matter intake, daily weight gain, and animal development (Noriega et al., 2008).

The implementation of coffee pulp silage in ruminants requires adequate handling of the pulp, avoiding its oxidation and the formation of fungi. It can be mixed with other raw materials such as sugar cane, urea, and molasses, allowing its conservation without affecting its nutritional composition and without representing a risk to the welfare of animals (Flórez & Rosales 2018).

In this context, it is important to include alternative ingredients in the diet of the animals that favor ruminal activity (Gallego et al., 2017a, b), increasing digestive efficiency and improving productive indicators in dairy cattle. Therefore, nutritional alternatives include coffee pulp silage (CPS), which allows adequate management of the waste generated in the agroindustry of this grain and minimizes feed costs (Yoplac et al., 2017).

Some studies carried out in tropical conditions have shown positive effects from the inclusion of coffee pulp in the diet of ruminants, when it was supplied as silage or a dehydrated form, replacing concentrate (Flórez, 2020), mainly feed supplements to dairy animals (Flórez & Rosales 2018).

We hypothesize that the inclusion and consumption of coffee pulp as DM in the diet for silage does not affect the productive, nutritional, and economic performance of crossbred milk cows. This research was conducted to evaluate the effects of the levels of inclusion of CPS in the diet on voluntary consumption, digestibility, metabolic profile, and the productive and economic performance of crossbred milk cows in the middle tropics.

## Materials and methods

### Location and weather conditions

The experiment was carried out in the Villa Marina Experimental Farm of the University of Pamplona, located in Matajira village, Pamplonita, Colombia, between August and December, corresponding to the rainy–dry transition season with a duration of 84 days. The experimental area is located in a mountainous region, with irregular topography at an altitude of 1100 m.a.s.l., with an average temperature and precipitation of 20 °C and 1400 mm, respectively.

### Animals, experimental design, and management

For this study, crossbred lactating cows (Holstein × Jersey × Swedish Red), multiparous in the lactation phase (the second third of lactation), with average initial body weight (BW) and age of 380 ± 10 kg and 6 ± 0.03 years, respectively, and body condition score of 3.8 on a scale of 1 to 5, were used according to Carizi et al. (2019).

The animals were distributed in a 4 × 4 Latin square design balanced for residual effect. The experiment consisted of four experimental subperiods of 21 days each. The first 14 days were used to adapt the animals to the experimental diets and area, and the last 7 days for sampling. The cows were kept in individual pens of approximately 10 m^2^ and provided with feeders and drinkers. In this way, the animals had unrestricted access to water and mineralized salt during the experimental period. The basal diet consisted of chopped *Pennisetum* sp*.* offered at 0600 h and 1700 h, allowing approximately 10% in orts, concentrate equal to 0.2% of the body weight (CP) during the milking (Table 1). The level of 0.2% of BW of concentrate (125 g CP day^−1^) accounted for approximately 15% of the requirements of crude protein (CP) for a crossbred cow, with 400 kg of CP and production of 7 kg day^−1^ of milk according to NRC (2001).

**Table 1:** Chemical composition of *Pennisetum* sp., coffee pulp silage, and concentrate consumed by the animals during the experimental period

Item	*Pennisetum* sp.	Coffee pulp silage	Concentrated
Dry matter (%)	15.38	19.82	87.00
Protein (%)	9.75	16.54	18.00
NDFap (%)	64.97	30.16	28.50
Ashes (%)	11.41	8.26	10.00
Ethereal extract (%)	1.58	1.71	3.00
NFC (%)	12.29	43.33	40.50
OM (%)	88.59	91.74	90.00

*NDFap* neutral detergent fiber corrected for ash and protein, *NFC* non-fiber carbohydrates, *OM* organic matter

The evaluated treatments were control (no CPS in the DM of the diet), 4.0% CPS included in the DM of the diet, 8.0% CPS included in the DM of the diet intake, and 12.0% CPS included in the DM of the diet intake. These inclusion levels correspond to 1.42, 2.84, and 4.26 kg of CPS in the natural matter and 0.28, 0.56, and 0.84 kg of dry matter for the 4%, 8%, and 12% inclusion of CPS of the dry matter of the diet, respectively. The CPS was supplied in its entirety in the morning hours and left available throughout the day for intake.

### Nutrient intake and digestibility

The voluntary intake and total digestibility were evaluated from the 15th day to the 22nd day of each experimental subperiod. To estimate the intake, the amount of feed offered (forage, CPS, and concentrate) and the orts of each animal were measured during this period. Finally, samples of food and orts were pooled per animal and experimental subperiod for subsequent analysis.

To estimate the excretion of fecal DM, total feces collections were carried out on days 16, 18, and 20 of each experimental subperiod for 24 h, starting at 0600 h. At the end of each day of collection, the feces were weighed. Subsequently, a sample of approximately 100 g was obtained, which was dried at 60 °C. Finally, samples were pooled per animal and experimental subperiod for subsequent analysis.

### Milk production and composition

The cows were milked daily at 0700 h using a milking machine (Moldel Km03061, Kurtsan, Istanbul, Turkey). However, to evaluate the production and composition of the milk, the milking was considered from the 16th day to the 21st day of each experimental subperiod. The milk was weighed immediately after milking, and then samples were collected in individual 50-mL bottles for later analysis. The exact time each cow was milked was recorded, and milk production over 24 h was calculated according to Almeida et al. (2020).

### Blood samples

On day 21 of each experimental subperiod, blood samples were collected to quantify the concentrations of glucose, cholesterol, triglycerides, serum urea nitrogen (SUN), albumin, globulin, and total protein. Samples were collected at 0700 h for glucose analysis via jugular venipuncture using vacutainer tubes with coagulation accelerator gel and vacuum tubes with sodium fluoride (BD Vacuntainer® SST II Advance, São Paulo, Brazil) and EDTA (BD Vacutainer ® Fluoride / EDTA, São Paulo, Brazil) as a glycolytic inhibitor and anticoagulant, respectively. The samples were immediately centrifuged at 3600 × g for 20 min, and the serum and plasma were stored at − 20 °C.

### Analytical procedures

The samples of forage, CPS, concentrate, and feces were analyzed according to the procedures described by Detmann et al. (2014) for DM (INCT-CA G-003/1), mineral matter (MM) (INCT-CA N-001/1), CP (INCT-CA M-001/1), ether extract (EE) (INCT-CA G-005/1), neutral detergent fiber (NDF) (INCT-CA F-002/1) with corrections for ash (CIDN) (INCT-CA M-002/1), and proteins (PIDN) (INCT-CA N-004/1).

The quantification of non-fibrous carbohydrates (NFC) was carried out according to Detmann and Valadares (2010):

$$\mathrm{NFC}=100-[\mathrm{\%CP}+\mathrm{\%NDFap}+\mathrm{\%EE}+\mathrm{\%MM}],$$

where CP is the crude protein; NDFap is the fiber in the neutral detergent corrected for ash and protein; EE is the ethereal extract; and MM is the mineral matter.

The milk yield is corrected for 4% fat using the following equation (NRC 2001):

$${\mathrm{Milk}}_{4\mathrm{\%}}(\mathrm{kg})\hspace{0.17em}=\hspace{0.17em}0.4\hspace{0.17em}\times \hspace{0.17em}(\mathrm{milk production})\hspace{0.17em}+\hspace{0.17em}[15\hspace{0.17em}\times \hspace{0.17em}(\mathrm{fat production}\hspace{0.17em}\times \hspace{0.17em}\mathrm{milk production}/100)]$$

Additionally, the milk was analyzed for lactose, fat, protein, density, and non-fat solids using a spectrophotometer (Lactoscan Julie C3 Scope Electric).

The concentrations of glucose (K082, Bioclin® Quibasa), triglycerides (K117, Bioclin® Quibasa), and total cholesterol (K083-2, Bioclin® Quibasa) in blood samples were quantified by enzymatic–colorimetric methods. The serum urea was analyzed by the enzymatic kinetic method (K056-1, Bioclin® Quibasa), and the total protein (K0311, Bioclin® Quibasa) and albumin (K040-1, Bioclin® Quibasa) were analyzed by colorimetric methods. The globulin concentrations were calculated as the difference between the total proteins and albumin levels analyzed. The SUN levels were estimated as 46.67% of the total serum urea. The blood parameters were analyzed in a commercial laboratory, following the manufacturer’s instructions using a biochemical analyzer (Mindray BS200E, Shenzhen, China).

### Benefit–cost ratio

To determine the benefit–cost ratio for each treatment, the costs and quantity of the feed supplement supplied were considered. To calculate the cost of the feed supplement, the raw material and the resources necessary for its production were taken into account. Thus, the relationship between the amount of milk produced and the amount of supplement consumed per treatment was established to estimate the productive efficiency of the animals. Finally, the profit was calculated from the income established in the region from sales per kilogram of milk.

### Statistical analysis

The experiment was analyzed according to a 4 × 4 Latin square balanced for residual effect, including treatment fixed effects and random effects of the animal and experimental period. All analytical procedures were performed using the PROC MIXED module of SAS 9.4 (SAS Institute Inc., Cary, NC, USA). Treatments were compared using orthogonal contrasts; contrasts were constructed to evaluate the effects of inclusion of CPS and the linear and quadratic effects of inclusion levels (4%, 8%, and 12%) in the DM of the diet. For the variables which did not present inclusion of the CPS effect but for which the linear or quadratic effect was significant, Dunnett’s test was performed to identify whether a supplemented treatment differed from the control treatment. Significance was considered at *P* < 0.05 and tendency when 0.05 < *P* ≤ 0.10.

## Results

### Forage samples, CPS, and concentrated

In this study, the forage, concentrated, and CPS intake by the animals presented a CP content of 9.75, 18.00, and 16.54%, respectively (Table 1).

### Nutrient intake and digestibility

The inclusion of CPS in the diet increases (*P* < 0.05) the voluntary intake in kg day^−1^ of DM, organic matter OM, CP, EE, NFC, digested OM (DOM), neutral detergent fiber (NDFcp), DNDF, and dietary NTD concentrations (Table 2). Additionally, an increasing trend (*P* = 0.083) in forage intake was verified with the inclusion of CPS in the diet compared to the control (Table 2). On the other hand, from the inclusion of 4% CPS, the forage intake linearly reduced (*P* < 0.05) with the increase in the inclusion of the levels of CPS. In contrast, the intake of CP and NFC in the animals linearly increased (*P* < 0.05) with increased levels of added CPS (Table 2). Additionally, an increasing trend was observed for the intake of OM (*P* = 0.080) and EE (*P* = 0.066) with increased inclusion levels of CPS in the diet (Table 2).

**Table 2:** Effect of the inclusion of CPS in the diet on the voluntary nutrient intake of crossbred milk cows

Ítem	Treatments				SEM	*P*-value		
	C	Inclusion level (%)						
		4.0	8.0	12.0		C vs. CPS	L	Q
kg/día								
DM	6.37	7.03	7.1	7.23	0.126	< 0.001	0.114	0.714
DMF	5.71	6.09*	5.87	5.73	0.126	0.083	0.015	0.714
DMC	0.66	0.66	0.66	0.66	-	-	-	-
CPSDM	-	0.28	0.54	0.84	-	-	-	-
OM	5.66	6.25	6.31	6.44	0.111	< 0.001	0.080	0.716
CP	0.68	0.76	0.78	0.82	0.012	< 0.001	0.001	0.694
EE	0.11	0.12	0.12	0.12	0.002	< 0.001	0.066	0.680
NDFap	3.9	4.23	4.17	4.16	0.082	0.002	0.397	0.715
NFC	0.97	1.14	1.23	1.34	0.015	< 0.001	< 0.001	0.738
DOM	2.63	3.35	3.40	3.54	0.140	< 0.001	0.179	0.692
NDFD	1.73	2.01	2.03	2.2	0.135	0.018	0.205	0.516
TDN	2.66	3.39	3.47	3.61	0.148	< 0.001	0.166	0.822
g/kg PC								
DM	16.78	18.53	18.7	19.05	0.372	< 0.001	0.122	0.726
DMF	15.04	16.04*	15.48	15.08	0.327	0.080	0.015	0.723
OM	14.89	16.46	16.64	16.70	0.331	< 0.001	0.099	0.733
NDFap	10.27	11.14	10.99	10.97	0.224	0.002	0.383	0.720

*DM* dry matter, *DMF* forage dry matter, *CDM* comercial feed matter, *CPSDM* coffee pulp silage dry matter, *OM* organic matter, *CP* crude proteín, *EE* ethereal extract, *FDNcp* neutral detergent fiber corrected for ash and protein, *NFC* non-fiber carbohydrates, *DOM* digested organic matter, *TDN* total digestible nutrients. C vs EPC: C vs S control versus supplementation; L and Q: effects of linear and quadratic order referring to the levels of inclusion of EPC in the diet. *Means statistically different from the control by Dunnett’s test

Regarding the voluntary intake in g kg^−1^ BW, an increase (*P* < 0.05) in the intake of DM, OM, and NDF was verified, and an increasing trend (*P* = 0.080) in the intake of DMF with the inclusion of CPS in the diet (Table 2). In contrast, from the treatment with 4% CPS included, the forage intake reduced linearly (*P* < 0.05) with increasing inclusion levels of CPS. Additionally, a linearly increasing trend (*P* = 0.099) was observed in the OM with increasing CPS inclusion levels in the diet (Table 2).

The inclusion of CPS increased (*P* < 0.05) the total digestibility of the DM, OM, and dietary DOM content (Table 3). Additionally, an increasing trend (*P* = 0.084) in NDF digestibility was observed with the inclusion of CPS in the diet (Table 3). There is also a linearly increasing trend (*P* = 0.084) in the NDF digestibility coefficient with increased CPS included in forage. However, a quadratic effect trend (*P* = 0.056) on the DM digestibility coefficient was verified with an increase in the CPS inclusion levels in the diet. However, no differences (*P* > 0.10) were observed for the other variables evaluated (Table 3).

**Table 3:** Effect of the inclusion of CPS in the diet on the total digestibility of nutrients of crossbred milk cows in the middle tropics

Ítem	Treatments				SEM	*P*-value		
	C	Inclusion level (%)						
		4.0	8.0	12.0		C vs. EPC	L	Q
g/g								
DM	0.418	0.489	0.496	0.515	0.0122	< 0.001	0.066	0.556
OM	0.465	0.537	0.540	0.550	0.0235	0.001	0.436	0.774
CP	0.335	0.426	0.368	0.359	0.057	0.282	0.229	0.558
EE	0.365	0.480	0.46	0.445	0.1488	0.643	0.475	0.559
NDFap	0.446	0.476	0.487	0.531	0.0367	0.084	0.121	0.563
NFC	0.692	0.783	0.834	0.737	0.0783	0.219	0.615	0.341
g/kg MS								
DOM	413	477	480	490	21.00	< 0.001	0.386	0.778

*DM* dry matter, *OM* organic matter, *CP* crude protein, *EE* ethereal extract, *NDFap* neutral detergent fiber corrected for ash and protein, *NFC* non-fiber carbohydrates, *DOM* digested organic matter. C vs CPS: C vs S control versus supplementation; L and Q: effects of linear and quadratic order referring to the levels of inclusion of EPC in the diet

### Milk production and quality

The production and chemical composition of milk was not affected (P > 0.10) by the inclusion of EPC in the diet (Table 4). However, a positive linear trend (P = 0.098) in lactose concentration with increased CPS inclusion levels in the diet was observed; in contrast, a negative linear trend (P = 0.062) was verified for the density of the milk with increased CPS inclusion. Additionally, a quadratic effect trend (P = 0.094) was present for the protein concentration in milk with increased CPS inclusion levels in the diet (Table 4).

**Table 4:** Effect of the inclusion of CPS in the diet on the production and chemical composition of the milk of crossbred dairy cows in the middle tropics

Ítem^1^	Treatments				SEM	*P*-value		
	C	Inclusion level (%)						
		4.0	8.0	12.0		C vs. CPS	L	Q
kg/día								
Milk prod	7.33	8.05	7.59	8.35	0.815	0.507	0.805	0.559
Milk prod. 4%	8.04	8.42	7.69	8.05	0.879	0.989	0.774	0.629
g/kg								
Lactose	40.6	46.6	40.5	42.1	1.992	0.238	0.098	0.100
Protein	28.1	32.1	28.0	30.8	1.490	0.227	0.553	0.094
Fat	41.8	40.6	40.3	33.5	3.280	0.369	0.178	0.448
SNG	75.0	85.8	74.6	77.6	3.63	0.242	0.097	0.101
g/mL								
Density	1.030	1.030	1.029	1.028	0.006	0.386	0.062	1.001

Milk prod.: daily milk production; Milk prod. 4%: fat daily milk production adjusted to 4% fat; SNG: non-fat solids. C vs CPS: C vs S control versus supplementation; L and Q: effects of linear and quadratic order referring to the levels of inclusion of CPS in the diet

### Blood metabolite concentration

In general, the inclusion of CPS in the diet did not influence (*P* > 0.10) the blood concentrations of the metabolites evaluated, only an increase (*P* < 0.05) in the blood albumin concentration was observed (Table 5). Additionally, a quadratic effect (*P* < 0.05) was verified for blood albumin concentrations with increasing levels of CPS in the diet (Table 5).

**Table 5:** Effect of the inclusion of CPS in the diet on the blood concentration of metabolites of crossbred dairy cows in the middle tropics

Ítem^1^	Treatments				SEM	P-value^2^		
	C	Inclusion level (%)						
		4,0	8,0	12,0		C vs. ICPS	L	Q
Glucose (mg dL^−1^)	59.15	60.59	59.92	59.11	0.754	0.4398	0.212	0.945
Cholesterol (mg dL^−1^)	169.39	169.87	169.55	169.69	0.264	0.364	0.761	0.309
Triglycerides (mg dL^−1^)	24.13	24.52	24.19	24.46	0.263	0.371	0.862	0.315
SUN (mg dL^−1^)	10.91	10.97	10.9	11.24	0.145	0.396	0.159	0.220
Total protein (g dL^−1^)	7.08	7.03	7.02	7.03	0.067	0.431	0.976	0.827
Albumin (g dL^−1^)	3.19	3.27	3.38	3.23	0.024	0.006	0.288	0.003
Globulins (g dL^−1^)	3.9	3.76	3.62	3.8	0.072	0.076	0.715	0.115

*SUN* sérum urea nitrogen. C vs CPS: C vs S control versus supplementation; L and Q: effects of linear and quadratic order referring to the levels of inclusion of CPS in the diet

### Economic analysis

The inclusion of CPS in the diet improved the productive efficiency of dairy cattle, which generated an increase in the profit from the commercialization of milk. Additionally, a decrease in the amount of supplement required (concentrate and EPC) was observed to produce 1 kg of milk with increased inclusion of EPC in the diet. However, increasing levels of EPC in the diet increased the cost of production per kilogram of milk produced (Table 6).

**Table 6:** Economic analysis

Ítem	Treatments			
	C	Inclusion level (%)		
		4.0	8.0	12.0
Cost of supplementation per kg milk produced (COP)	189.25	185.99	238.46	254.54
Profit per milk marketed (COP)	468,409.20	516,622.51	453,052.99	487,793.71
Difference between treatments in relation to control	-	10.29	− 3.27	4.13
Supplement/milk ratio	8.98	4.28	2.43	1.90
Benefit:cost	3.01:1	3.08:1	2.18:1	1.98:1

C vs EPC: C vs S control versus supplementation; L and Q: effects of linear and quadratic order referring to the levels of inclusion of EPC in the diet

## Discussion

Studies of cattle in tropical conditions indicate the positive effects of supplementation with nitrogen compounds and multiple supplements on the voluntary intake of forage, which is maximized when the CP content in the diet is 14.5% CP kg^−1^ DM (Detmann et al., 2014). In this study, the content of dietary CP was 10.68 and 11.10 PB/kg DM for the control treatments and those that received additional CPS in the diet. These values are lower than those recommended by Detmann et al. (2014); however, a higher concentration of CP was observed in the diets that included CPS. This may explain the increase in intake in kg day^−1^ and g kg^−1^ of DM, DMF, NDFap, and NDFD in the animals that received CPS (Table 2). Similar results have been obtained by Sotelo et al. (2018) and Franco et al. (2021) when they supplemented animals fed with tropical forage and by Oliveira et al. (2007) when substituting corn silage for coffee hulls.

The higher intake of CP, EE, NFC, and OM of the animals that received CPS in the diet compared to the control group can be justified by the higher concentration of these components in the CPS compared to forage (Table 1). This difference may also explain the linear increase in the intake of CP, EE, and NFC of the animals with the increased levels of included CPS in the diet. This behavior promoted a higher intake of DOM and TDN in the animals with added CPS in their diets (Table 2).

For Machado et al. (2011), the digestibility of the diet was the result of the interactive and associative effects of all the dietary components instead of the isolated effect of a certain component. The higher DM and OM digestibility coefficient of the groups with added CPS intake relative to the control group (Table 3) seem to be directly associated with the higher DM intake of these animals (Table 2), which decreases the representativeness of the fecal metabolic fraction of DM and OM. Additionally, the higher digestibility of NDFap in the animals that received added CPS can be explained by the higher intake of CP in these animals, reinforcing the benefits of supplementation with positive compounds on the digestibility of fibrous carbohydrates by ruminal microorganisms. However, the trend toward a linear increase in DM intake in animals that received CPS may be due to the increased DM intake with increased levels of CPS in the diet (Table 2).

Despite the higher intake of CP, EE, and NFC by the animals that received CPS compared to the control group (Table 2), there was no evidence of a higher digestibility coefficient of these components in the animals. This may be due to the intake of concentrate, which is easily digested and increases the digestibility of dietary components. Although the increase in the OM digestibility coefficient and the dietary content of MOD in the animals that received CPS seems to be directly related to the increase in the digestibility of NDFap, the digestibility of CP, EE, and NFC were not affected (Table 3).

In general, feeding animal supplements generates an increase in their productive performance; however, this effect may be limited by the protein and energy content of the supplement (Castillo & Domínguez, 2019) and by imbalances in the protein–nitrogen energy ratio that enters the rumen (Gutiérrez et al., 2017). In this study, although a higher intake of DM, OM, CP, NFC, and NDFap was evident in animals that received CPS (Table 2), no effect on milk production was verified (Table 4), which indicates that the higher intake of DM, CP, NFC, EE, NFD, and OM for the animals that received CPS was not enough to impact production. However, an increasing trend was observed in the concentration of lactose and NGS in the milk produced (Table 4). This increase in the concentration of lactose and NGS can be justified by the higher intake of DM, OM, CP, and NFC of these animals, which promotes an increase in the production of propionate at the rumen, which is metabolized to glucose in the liver, and therefore, higher lactose synthesis occurs in the mammary gland (Huntington et al., 2006). This behavior may explain the observed increase in NGS concentration.

In contrast, the quadratic trend in the concentration of protein in milk with increased CPS in the diet (Table 4) observed in this study, which presented its lowest value when 4% CPS was included in the diet, can be justified by the compounds present in CPS, such as polyphenols and caffeine, which negatively affect the ruminal degradation of protein and its efficient use for metabolic processes (Flórez & Rosales, 2018). The milk composition values obtained in this study are similar to those reported by Valencia (2013), who supplemented milk cows under tropical conditions. Similar results were obtained by Rocha et al. (2006) who used coffee pulp in a proportion of 10% of the cattle diet without showing an effect on milk quality. Additionally, Barragán et al. (2019) classified milk according to its composition; thus, milk is considered normal when it has a fat concentration higher than 36.4 g kg^−1^, and the concentrations of protein and solids are 30.8 and 106.0 g kg^−1^ of milk, respectively. In this study, the fat and protein values were 39.05 and 29.75 g kg^−1^ of milk, being values ​​very close to those reported by Barragán et al. (2019).

The concentrations of glucose, cholesterol, and triglycerides in the blood are important indicators of energy metabolism and nutritional status of animals, especially lactating animals, allowing the evaluation of the energy intake for milk production (Gallego et al., 2017a, b). Despite the higher intake of DM, CP, NFC, and OM for the animals that received CPS (Table 2), no difference was found in glucose, cholesterol, and triglyceride concentrations between treatments (Table 5). This indicates that the supplied diets promoted a similar energy status in the animals.

According to Van Soest (1994), the concentration of urea in the blood is positively associated with CP intake. However, the absence of an effect on the NUS between treatments, despite the higher intake of CP by the animals that received CPS, was an unexpected result, which may indicate more efficient use of nitrogen compounds in the rumen by the animals of the control group.

Albumin, globulins, and total proteins are produced in the liver; their synthesis is related to the intake of CP, amino acids, and nutrients (Lawrence et al., 2012). These indicate the nutritional status and protein metabolism of animals. Thus, a higher concentration of albumin and the tendency to increase blood globulin in the animals that received CPS and the absence of an effect on total proteins can be explained by the higher intake of CP by these animals, showing that albumin and the globulins are more sensitive indicators of the protein status of animals compared to total proteins (Herrera et al., 2018). Additionally, a higher albumin concentration in the blood was verified when 4% CPS was included in the diet.

The cost of supplementation per kilogram of milk produced with CPS was higher than conventional supplementation with concentrate. The inclusion of 4% CPS in the DM of the diet of dairy cows obtained 10.29% more gains than the exclusive use of concentrate. The CPS reduced the number of supplements required to produce 1 kg of milk. This same treatment presented the best benefit:cost ratio, 3.08. This situation is a reflection of the national availability of this by-product of the coffee agroindustry and its simple processing that facilitates its storage without affecting its nutritional quality, making it possible to use it from a technical, environmental, and economic point of view in cattle farms.

The inclusion of 4% CPS in the dry matter of the diet is a nutritional strategy that improves the nutritional characteristics and the concentration of albumin and globulins in the blood of crossed dairy cows in the middle tropics. However, it does not improve the milk production of the animals. Additionally, the amount of supplement (concentrate and CPS) per kilogram of milk produced is improved, generating a positive effect on the profit margin and taking advantage of the residue from coffee production, which benefits the environment.

## Data Availability

The data sets generated and/or analyzed during the current study are not publicly available as they are the intellectual property of the American University of Europe UNADE, but are available from the corresponding author upon reasonable request.
